# Efficacy of Aortic Valve Replacement through Full Sternotomy and Minimal Invasion (Ministernotomy)

**DOI:** 10.3390/medicina54020026

**Published:** 2018-04-28

**Authors:** Hammad M. A. Aliahmed, Rimantas Karalius, Arūnas Valaika, Arimantas Grebelis, Palmyra Semėnienė, Rasa Čypienė

**Affiliations:** Department of Cardiovascular Medicine, Vilnius University, 01513 Vilnius, Lithuania; rimantas.karalius@santa.lt (R.K.); arunas.valaika@santa.lt (A.V.); arimantas.grebelis@santa.lt (A.G.); palmyra.semeniene@santa.lt (P.S.); rasa.cypiene@santa.lt (R.Č.)

**Keywords:** aortic valve replacement, ministernotomy, sternotomy

## Abstract

*Background:* new minimally invasive sternotomy (mini-sternotomy) procedures have improved the treatment outcome and reduced the incidence of perioperative complications leading to improved patient satisfaction and a reduced cost of aortic valve replacement in comparison to the conventional median sternotomy (full sternotomy). The aim of this study is to compare and gain new insights into operative and early postoperative outcomes, long-term postoperative results, and 5-year survival rates after aortic valve replacement through a ministernotomy and full sternotomy. *Methods:* This is a retrospective study of patients who underwent an isolated replacement of the aortic valve via a full sternotomy or ministernotomy from 2011 to 2016. From 2011 to 2016, 426 cardiac interventions were performed, 70 of which (16.4%) were of the ministernotomy and 356 (83.6%) of the full sternotomy. Through propensity score matching, 70 patients who underwent the ministernotomy (ministernotomy group) were compared with 70 patients who underwent the full sternotomy (control group). *Results:* in the propensity matching cohort, no statistical difference in operative time was noted (*p* = 0.856). The ministernotomy had longer cross clamp (88.7 ± 20.7 vs. 80.3 ± 24.6 min, *p* = 0.007) and bypass (144.0 ± 29.9 vs. 132.9 ± 44.9 min, *p* = 0.049) times, less ventilation time (9.7 ± 1.7 vs. 11.7 ± 1.4 h, *p* < 0.001), shorter hospital stay (18.3 ± 1.9 vs. 21.9 ± 1.9 days, *p* = 0.012), less 24-h chest tube drainage (256.2 ± 28.6 vs. 407.3 ± 40.37 mL, *p* < 0.001), fewer corrections of coagulopathy (*p* < 0.001), fewer patients receiving catecholamine (5.71 vs. 30.0%, *p* < 0.001) and better cosmetic results (*p* < 0.001). Moreover, the number of patients without complaints at 1 year after the operation was significantly greater in the ministernotomy group (*p* = 0.002), and no significant differences in the 5-year survival between the groups were observed. In the overall cohort, the ministernotomy had longer cross clamp times (88.7 ± 20.7 vs. 79.9 ± 24.8 min, *p* < 0.001), longer operative times (263.5 ± 62.0 vs. 246.7 ± 74.2 min, *p* = 0.037) and bypass times (144.0 ± 29.9 vs. 132.7 ± 44.5 min, *p* = 0.026), lower incidence of 30-day mortality (1(1.4) vs. 13(3.7), *p* = 0.022), shorter hospital stays post-surgery *p* = 0.025, less 24-h chest tube drainage, *p* < 0.001, and fewer corrections of coagulopathy (*p* < 0.001). *Conclusions:* the ministernotomy has a number of advantages compared with the full sternotomy and thus could be a better approach for aortic valve replacement.

## 1. Introduction

Recently, the number of cases with aortic pathology has increased markedly, which has resulted in the search for conservative surgical treatment methods [1,2,3]. Consequently, new minimally invasive approaches (e.g., upper and lower ministernotomy; V-shaped, Z-shaped, T-shaped, J-shaped sternotomy; and other types of ministernotomy) have been introduced [4,5,6]. New ministernotomy techniques have improved the treatment and reduced the incidence of perioperative complications compared with the full sternotomy [2,3,4,5,6].

Currently, a number of studies comparing short-term and long-term results of full sternotomy and minimally invasive techniques have been conducted for hemisternotomy [7,8,9]. Minimally invasive approaches reduced the amount of blood loss, probability of infection and hospitalization duration; improved cosmetic results; and accelerated patient recovery [10,11,12,13]. Despite the numerous advantages inherent to minimally invasive techniques, some authors have noted several negative effects, such as longer aortic cross-clamp and cardiopulmonary bypass times [12,13,14], which could in turn influence surgery performance and could be an unfavorable factor for patients of advanced age [15,16]. Moreover, some studies have explored short-term survival outcomes of minimally invasive techniques as compared with those of full sternotomy. Byrne et al. and Tabata et al. suggest that ministernotomy limits the invasiveness of surgical interventions, which may reduce morbidity [17,18]. They argue that ministernotomy avoids unnecessary lower mediastinal dissection leading to reduced blood loss, need for transfusion, and length of stay in the critical care unit and in the hospital. Using a larger series of procedures (1126 patients), Totaro et al. investigated the perioperative mortality arising from re-do procedures, isolated first-time aortic valve replacement, and complex procedures. Their findings demonstrated that minimal invasion may be particularly useful in patients undergoing complex re-do surgical procedures [19].

In this study, we present our experience with ministernotomy. The aim of our study was to analyze and compare operative, early postoperative outcomes, long-term postoperative results and 5-year survival after aortic valve replacement through ministernotomy and full sternotomy. The study is unique in the sense that we perform a comprehensive comparison of the outcomes of the two procedures over a longer period than previous studies and all the possible influences of the type of access on the patient’s health.

## 2. Materials and Methods

This is a retrospective study of patients who underwent isolated aortic valve replacement through sternotomy or ministernotomy. All operations were performed between 1 October 2011 and 1 January 2016 in the department of cardiovascular medicine, Vilnius University Hospital Santaros Klinikos. From 2011 to 2016, 426 cardiac interventions were performed, of which 70 (16.4%) were minimal access surgery and 356 (83.6%) were interventions by means of a longitudinal sternotomy. Permission to conduct the study was obtained from Vilnius Regional Biomedical Research Ethics Committee (No. 158200-14-715-235).

The patients were selected according to age, sex, body mass index, etiology of the underlying disease, diagnosis, New York Heart Association (NYHA) evaluation, and echocardiographic parameters. To reduce selection bias, we used propensity score matching. Both the ministernotomy and full sternotomy (control) groups had 70 patients each. Patients requiring reoperation and procedures such as coronary artery bypass grafting, surgery of mitral or other valves, ascending aorta replacement, atrial fibrillation ablation, or aortic valve plasty were excluded from the study.

### Statistical Analysis

Evaluation of dichotomous variables was done using the Fisher exact test, and the data are presented as percentages and number of cases. Categorical data were analyzed using the chi-square test. Continuously distributed variables were evaluated using the Student t test and presented as the mean ± standard deviation. The Shapiro–Wilk test was applied to check the normality of distribution of our data. Five-year survival was evaluated using Kaplan–Meier analysis and compared statistically using the log rank test. To reduce selection bias, a propensity score was calculated for each patient by logistic regression. A multinomial propensity score-matched cohort was constructed by nearest neighbor matching without replacement. Statistical analysis were performed using the Statistical Package for Social Sciences, version 21.0 (SPSS, Chicago, IL, USA), and *p* < 0.05 was considered statistically significant.

## 3. Results

The number of males was slightly higher than that of females in full sternotomy and ministernotomy (54.2% and 60%, respectively). Overall, the sternotomy patients were slightly older with a mean age of 61.04 ± 11.9 years than the ministernotomy patients with a mean of 60.8 ± 11.4 years (*p* = 0.155). However, the difference in age of patients in full sternotomy and ministernotomy was not significant. Table 1 illustrates the significant preoperative characteristics for the minimal invasive and full sternotomy patients. The characteristics between the groups were similar before surgery; however, EuroSCORE II >3% occurred more frequently in the sternotomy group than in the ministernotomy group (*p* = 0.006 in the overall cohort, *p* = 0.049 in the propensity matched cohort). A majority of patients were NYHA Class III in both conventional sternotomy as well as in ministernotomy in both cohorts. There were 81.43% NYHA Class III in the mini-sternotomy group and 84.6% in the sternotomy group in the overall cohort. In the propensity score matched cohort, there were 81.43% in the ministernotomy group and 91.4% in the sternotomy group. Analysis of comorbidity showed that the ministernotomy group had more patients with hypertension than the sternotomy group (*p* = 0.003, *p* = 0.016 respectively). Chronic obstructive pulmonary disease (COPD) was significantly more frequent in patients with minimally invasive access (4 (5.7%), *p* = 0.042); COPD was not observed in the full sternotomy group.

Table 2 presents the intraoperative characteristics of both groups. In the overall cohort, a statistical difference in operative time was noted (*p* = 0.037). Ministernotomy had longer cross clamp (88.7 ± 20.7 vs. 79.9 ± 24.8 min, *p* < 0.001) and bypass (144.0 ± 29.9 vs. 132.7 ± 44.5 min, *p* = 0.026) times. Biological and mechanical valve type were used in the ministernotomy and sternotomy. There was a significant difference in the number of patients in whom a biological or mechanical valve was used in both the overall cohort and PSM cohort (*p* < 0.001).

In the propensity matched cohort, no statistical difference in operative time was noted (*p* = 0.856). The ministernotomy required significantly longer aortic cross-clamping time (8 min longer) than that required for the sternotomy (*p* = 0.007). The ministernotomy also required longer cardiopulmonary bypass time, which lasted almost 12 min longer than that in the full sternotomy (*p* = 0.049). Repeated cardioplegia was performed in 3 (4.3%) patients from the sternotomy group, but was not applied to the ministernotomy group (*p* = 0.080).

Postoperative statistics (Table 3) show that the ministernotomy had a few advantages over the full sternotomy. In the overall cohort, there was a lower incidence of 30-day mortality, of 1.4% in the ministernotomy group and 3.7% in the full sternotomy group (*p* = 0.022), a lower incidence of repeated cardioplegia, *p* = 0.042, shorter hospital stay post-surgery (17.6 ± 16.9 vs.13.06 ± 1.0 *p* = 0.025), and less 24-h chest tube drainage, *p* < 0.001.

In the propensity matched cohort, the duration of mechanical ventilation was significantly shorter during the ministernotomy than the full sternotomy (9.7 ± 1.7 h vs. 11.7 ± 1.4 h, *p* = 0.001). The amount of blood flowing through the chest tube drainage in the ministernotomy was almost 1.5 times more than that in the full sternotomy (*p* < 0.001). The total duration of hospitalization in the full sternotomy group was almost 4 days longer than that of the ministernotomy group (*p* = 0.012). Fourteen patients (20%) in the full sternotomy group underwent a correction of coagulopathy; no correction of coagulopathy was performed in the ministernotomy group (*p* < 0.001). Moreover, an evaluation of drug therapy for hemodynamic support showed that catecholamine is used significantly more often in the full sternotomy group than in the ministernotomy group (30.0% vs. 5.71%, *p* < 0.001). Evaluation of the use of pain medication showed that morphine is used significantly more often during the full sternotomy than during the mini-sternotomy (98.57% vs. 90.00%, *p* = 0.029). Stroke in both groups was not observed after surgery.

The analysis of factors predicting mortality after aortic valve replacement have shown that there is a strong correlation between left ventricular ejection fraction (LVEF) and the requirement of correction for coagulopathy. Patients with LVEF between 30–49% and with need for correction of coagulopathy have high chances of death as compared to patients with LVEF between >50% and with no requirement for correction of coagulopathy, as shown in Table 4.

**Figure 1:**
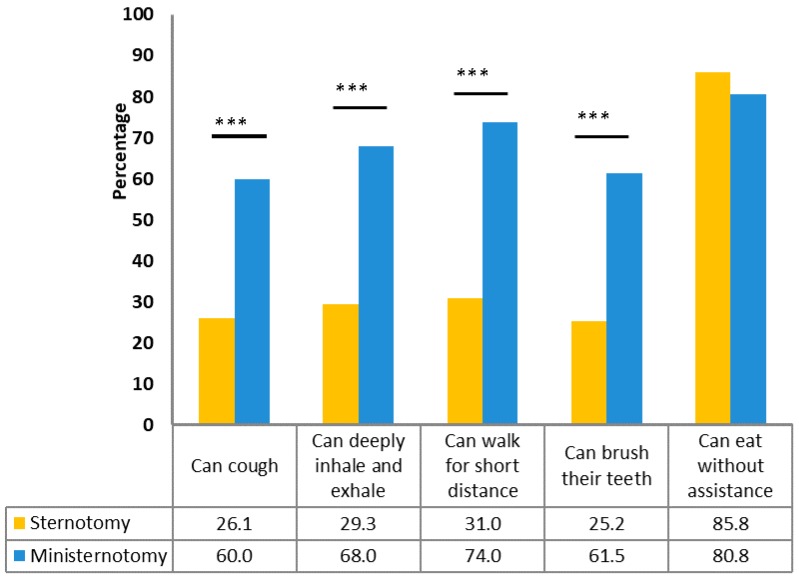
Effect of surgery on post-surgery quality of life in the overall cohort (in the first 7 days after surgery). *** *p* < 0.001.

**Figure 2:**
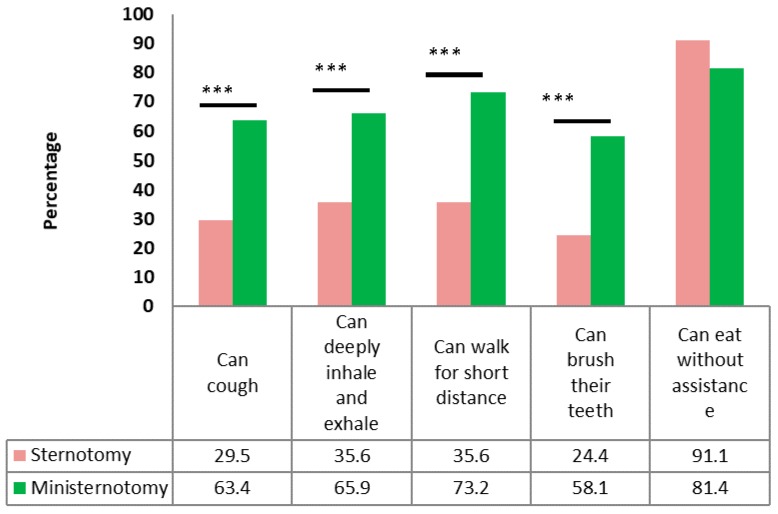
Effect of surgery on post-surgery quality of life in the propensity matching cohort (in the first 7 days after surgery). *** *p* < 0.001.

**Figure 3:**
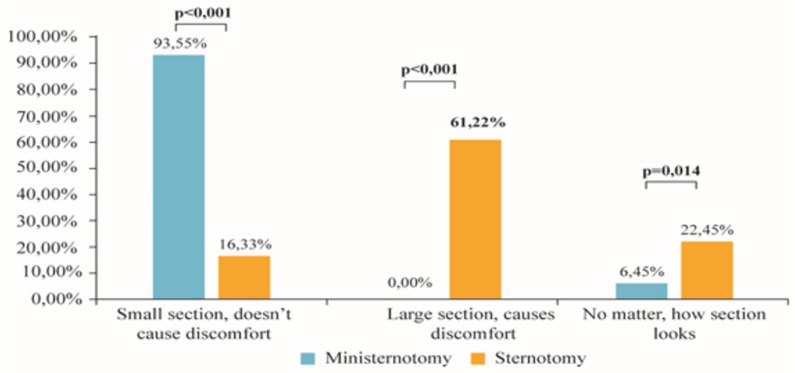
Survey into the cosmetic effects of both methods in the propensity matching cohort.

Based on a patient satisfaction survey, a significant increase in satisfaction after the ministernotomy was found, which could be attributed to a faster return to activities of daily living and better cosmetic results (Figure 1, Figure 2 and Figure 3). Except for eating, there was a higher percentage of patients with mini-sternotomy surgery compared to full sternotomy patients, who can cough, breathe, walk a short distance and brush their teeth by themselves. There were no patients with a surgical wound in the ministernotomy, while most patients in the sternotomy group felt discomfort because of their postoperative wounds. The results of the survey about the cosmetic effects of both methods in the propensity matching cohort are shown in Figure 3.

To evaluate the long-term results of the operations, echocardiographic parameters, NYHA stages, and clinical symptoms were examined for 3 years. After 1 year, the number of patients without complaints was significantly greater in the ministernotomy group (*n =* 61) 60 (98.4%) than in the full sternotomy group (*n =* 57) 46 (80.7%) (*p* = 0.002). During this period, no new cardiac interventions were performed, and on the second and third year of observation, significant differences in echocardiographic parameters, NYHA stage, and clinical symptoms between groups were not observed.

**Figure 4:**
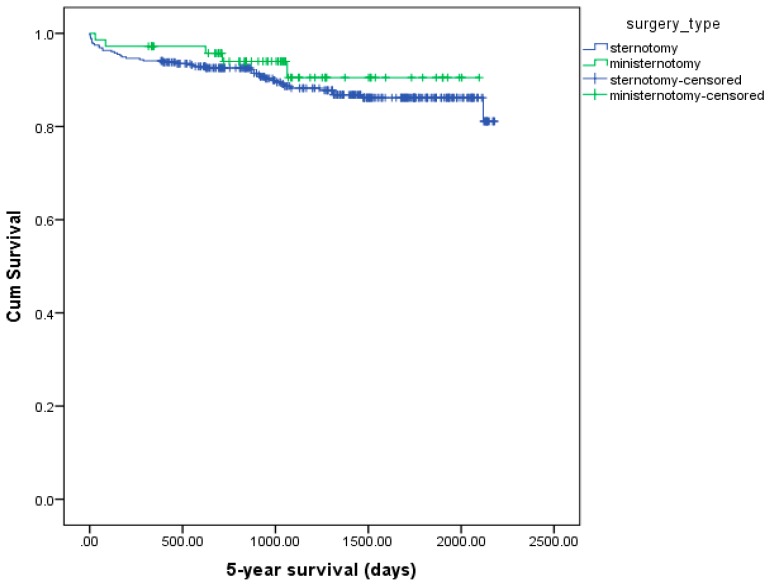
Kaplan–Meier curves; 5-year survival curve of overall cohort.

**Figure 5:**
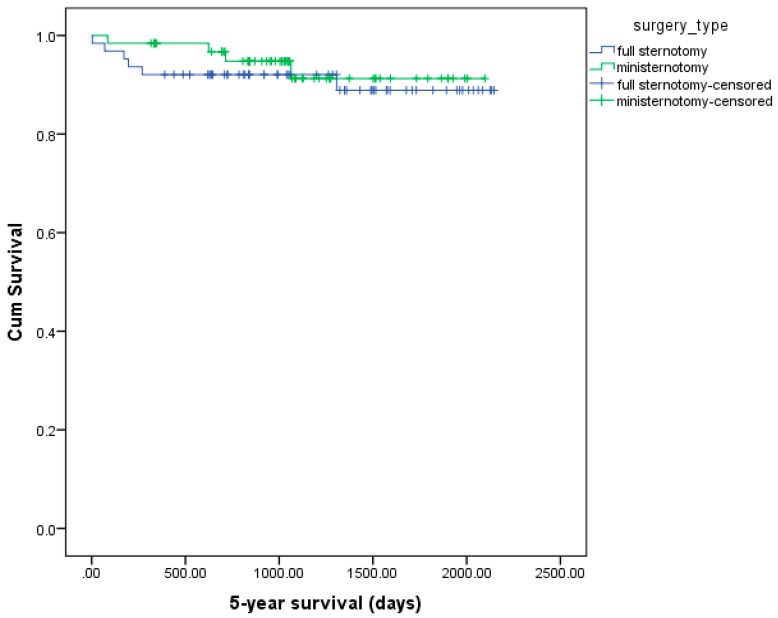
Kaplan–Meier curves; 5-year survival curve of propensity matching cohort.

Thus, the major differences between the two operational techniques were observed in the early postoperative period. We found that the differences between groups decreased in the long term. Moreover, our findings were confirmed by the analysis using the Kaplan–Meier methodology (Figure 4 and Figure 5). In the overall cohort, the 5-year survival of the ministernotomy group was 91.3% while that in the full sternotomy group was 97.4% (*p* = 0.582) Figure 4. In the propensity matched cohort, the 5-year survival: 66 (94.3%) in the ministernotomy group, 67 (95.7%) in the sternotomy group, total 133 (95%). The log-rank test revealed no significant differences in survival between the groups (*p* = 0.659) Figure 5.

## 4. Discussion

In this study, the evaluation of the results of the full sternotomy and the ministernotomy for aortic valve replacement revealed that the duration of the ministernotomy is longer than that of the full sternotomy. However, no statistical difference was observed in the propensity score matched cohort. We found that the ministernotomy requires a longer time to clamp the aorta during surgery and a longer cardiopulmonary bypass time. These results are consistent with previous findings [10]. Glauber et al. [9] and Lim et al. [14] reported that aortic valve replacement via a ministernotomy could be performed safely, despite the longer ischemic time. This fact is confirmed in the works of the majority of authors, which can worsen the parameters of the operation as a whole, and also become a particularly unfavorable factor for elderly patients. Nevertheless, opposite favorable tendencies were reported in some studies. In a retrospective analysis of the treatment of 2103 patients who underwent primary, isolated aortic valve replacement (ministernotomy approach (*n =* 936); full sternotomy approach (*n =* 1167)), Shehada et al. demonstrated that the time of aorta clamping during the operation was not statistically different between the full sternotomy and ministernotomy [11]. Their study showed that 30-day mortality for the ministernotomy and full sternotomy are not significantly different; their study advocated that the ministernotomy is a safe and effective procedure associated with a low mortality rate and good long-term survival rates. In addition to that, the ministernotomy was associated with shorter ventilation times, a lower rate of autologous blood transfusion, as well as a lower rate of postoperative respiratory and renal insufficiency.

When assessing postoperative indices, we found that the ventilation time during the ministernotomy was significantly shorter than that during the full sternotomy, which is also comparable with the results of other studies [20,21]. Moreover, we noted a smaller amount of bleeding in the first 24 h after surgery through the chest tube drainage in the ministernotomy group. In the work of Yilmaz et al. [21], the amount of blood loss was also significantly less during the ministernotomy. After a ministernotomy, a significantly smaller number of patients needed medical correction of coagulopathy. Hence, the small amount of blood volume loss during the ministernotomy not only could contribute to a more rapid patient recovery but also has a significant economic implication, i.e., in relation to the medications for the correction of coagulopathy.

The duration of hospitalization was almost 4 days longer in the full sternotomy group. Similar results are found in several studies comparing the ministernotomy and full sternotomy [10,12,21]. A shorter hospitalization duration is one of the main advantages of most minimally invasive methods. Furthermore, a comparison of the frequency of postoperative complications, such as postoperative wound infections, atrial fibrillation, cardiac tamponade, embolism, and acute renal failure, revealed no significant differences between the groups. Similar results were found in a previous meta-analysis [9].

Based on the survey, patient satisfaction with the ministernotomy increased, which could be attributed to a faster return to activities of daily living and better cosmetic results. Several authors have demonstrated an improved cosmetic appearance of the postoperative wound after a ministernotomy [9,11,12]. Nevertheless, patient satisfaction in relation to a small incision lies not only in the better postoperative wound appearance but also in decreased pain intensity during breathing and the activities of daily living. Amr [12] also wrote about this in his study, and noted that in the early hospital period patients after a J-shaped upper ministernotomy could freely breathe without restrictions to their physical activity. Another important advantage of a small incision is the prevention of sternal wound dehiscence or overriding edges. A smaller wound contributes to a more rapid patient recovery [9]. Moreover, a year after the operation, the number of patients without complaints associated with their underlying disease and surgical intervention was significantly higher in the ministernotomy group; however, during the follow-up at 3 years, the difference was no longer statistically significant.

According to the results obtained in our study over a 5-year period, the differences between the groups were no longer statistically significant in the long term, which was confirmed by the Kaplan–Meier analysis. No significant differences in the 5-year survival between the groups were observed. Similar results were obtained by Skripochnik et al. [22]; in assessing 1- and 4-year survival, they noted no significant differences between patients in the mini-invasive and those in full access groups. However, Merk et al. reported higher 5- and 8-year survival rates in patients who underwent minimal access operation than in those who underwent traditional interventions [20]. Although the authors could not explain this unique finding, they suggested that it could be due to the fact that the surgeons in the institution under investigation had more experience in performing surgery by minimal access.

This study has some limitations: the retrospective nature of the study, the single center experience, the limited number of study patients, and the fact that preoperative matching between the two groups could have been assessed only with larger groups. Nevertheless, preoperative characteristics were comparable between group ministernotomy and group sternotomy. Another limitation in this study was the lack of standardized recommendations for discharging patients after ministernotomy surgery.

## 5. Conclusions

Based on our findings, aortic valve replacement with the minimally invasive approach can be sufficiently safe and effective. Surgeries with the said approach reduce the duration of hospitalization, ventilation time, blood loss, surgical trauma and corrections of coagulopathy, improve cosmetic results, and speed up patient rehabilitation and the resumption of normal activities.

## Figures and Tables

**Table 1:** Preoperative patients’ characteristics.

Indicator	Overall Cohort			Propensity Score Matched Cohort		
	Mini-Sternotomy *n =* 70	Full Sternotomy *n =* 356	*p*	Mini-Sternotomy *n =* 70	Full Sternotomy *n =* 70	*p*
Age, mean ± SD, years	60.8 ± 11.6	63.3 ± 13.2	0.155	60.8 ± 11.6	61.4 ± 11.9	0.155
Male, *n* (%)	42 (60.0)	193 (54.2)	0.384	42 (60)	42 (60)	1.000
Height, mean ± SD, cm	170.8 ± 10.0	169.3 ± 10.2	0.247	170.8 ± 10.0	170.1 ± 9.0	0.247
Body weight, mean ± SD, kg	82.3 ± 1.8	82.6 ± 1.8	0.886	82.3 ± 1.8	81.0 ± 1.6	0.886
BMI, mean ± SD, kg/m^2^	27.7 ± 4.4	28.8 ± 5.6	0.656	27.7 ± 4.4	27.9 ± 4.5	0.536
Diagnosis, *n* (%)						
Aortic stenosis	51 (72.9)	267 (75.2)	0.822	51 (72.9)	45 (64.3)	0.754
Aortic regurgitation	12 (17.1)	50 (14.1)	0.502	12 (17.1)	13 (18.6)	0.825
Combined	7 (10.0)	38 (10.7)	0.763	7 (10.0)	12 (17.1)	0.217
Etiology of the disease, *n* (%)						
Senile degeneration	53 (75.71)	283 (80.4)	0.350	53 (75.71)	53 (75.71)	1.000
Annular expansion	13 (18.6)	47(13.4)	0.238	13 (18.6)	12 (17.14)	0.825
Mitral valve	1 (1.4)	0 (0.0)	0.024	1 (1.4)	0 (0.0)	0.316
Infective endocarditis	3 (4.3)	22 (6.3)	0.538	3 (4.3)	5 (7.14)	0.466
EuroSCORE II						
<1%, *n* (%)	24 (34.3)	99 (27.8)	0.253	24 (34.3)	20 (28.6)	0.466
1–3%, *n* (%)	44 (62.9)	201 (56.5)	0.344	44 (62.9)	42 (60.0)	0.728
>3%, *n* (%)	2 (2.9)	56 (15.7)	0.004	2 (2.9)	8 (11.4)	0.049
NYHA functional class, *n* (%)						
II	12 (17.14)	41 (11.5)	0.192	12 (17.14)	6 (8.6)	0.130
III	57 (81.43)	301 (84.6)	0.514	57 (81.43)	64 (91.4)	0.084
IV	1 (1.4)	14 (3.9)	0.299	1 (1.4)	0 (0.0)	0.316
Creatinine clearance (mL/min)						
<50, *n* (%)	41 (58.6)	3 (0.8)	0.144	41 (58.6)	36 (51.4)	0.396
50–85, *n* (%)	27 (38.6)	196 (55.4)	0.075	27 (38.6)	31 (44.3)	0.493
>85, *n* (%)	2 (2.86)	155 (43.8)	0.098	2 (2.86)	3 (4.3)	0.649
Diabetes mellitus, *n* (%)	11 (15.7)	5 (7.1)	0.111	11 (15.7)	5 (7.1)	0.111
COPD, *n* (%)	4 (5.7)	0 (0.0)	0.042	4 (5.7)	0 (0.0)	0.042
Hypertension, *n* (%)	22 (31.4)	57 (16.1)	0.003	22 (31.4)	10 (16.0)	0.016
Stroke, *n* (%)	0 (0.0)	16 (4.5)	0.086	0 (0.0)	2 (2.9)	0.154
Coronary artery disease, *n* (%)	3 (4.3)	12 (3.4)	0.723	3 (4.3)	1 (1.4)	0.310
Renal failure, *n* (%)	2 (2.9)	1 (1.4)	0.5590.000	2 (2.9)	1 (1.4)	0.559
Pacemaker, *n* (%)	5 (7.1)	16 (4.5)	1.000	5 (7.1)	2 (2.9)	0.245
LVEF %, *n* (%)						
<30%	0 (0.0)	0 (0.0)	ND	0 (0.0)	0 (0.0)	ND
30–49%	16 (22.9)	11 (15.7)	0.580	16 (22.9)	11 (15.7)	0.284
≥50%	54 (77.1)	59 (84.3)	0.826	54 (77.1)	59 (84.3)	0.284

LVEF, left ventricular ejection fraction; COPD, chronic obstructive pulmonary disease; NYHA, New York Heart Association Functional Classification; BMI, body mass index; EuroSCORE II, European System for Cardiac Operative Risk Evaluation, version II. ND, not determined.

**Table 2:** Characteristics of surgery in the two groups.

Indicator	Overall Cohort			Propensity Score Matched Cohort		
	Full Sternotomy *n =* 356	Mini-Sternotomy *n =* 70	*p*	Full Sternotomy *n =* 70	Mini-Sternotomy *n =* 70	*p*
Surgery duration, mean ± standard deviation (SD), min	246.7 ± 74.2	263.5 ± 62.0	0.037	256.9 ± 79.7	263.5 ± 62.0	0.856
Aortic cross-clamping time, mean ± SD, min	79.9 ± 24.8	88.7 ± 20.7	<0.001	80.3 ± 24.6	88.7 ± 20.7	0.007
Cardiopulmonary bypass time, mean ± SD, min	132.7 ± 44.5	144.0 ± 29.9	0.026	132.9 ± 44.9	144.0 ± 29.9	0.049
Repeated cardioplegia, *n* (%)	11 (3.1)	0 (0.0)	0.224	3(4.3)	0 (0.0)	0.080
Valve type, *n* (%)						
Biological	163 (45.8)	60 (85.7)	<0.001	24 (34.3)	60 (85.7)	<0.001
Mechanical	193 (54.2)	10 (14.3)	<0.001	46 (65.7)	10 (14.3)	<0.001
Size of aortic valve prosthesis, *n* (%)						
19 mm	1 (0.3)	0 (0.0)	0.657	0 (0.0)	0 (0.0)	ND
21 mm	57 (16.0)	4 (5.6)	0.025	7 (10.0)	4 (5.7)	0.346
23 mm	154 (43.3)	34 (47.2)	0.549	36 (51.4)	33 (47.1)	0.612
25 mm	111 (31.2)	30 (41.7)	0.095	19 (27.1)	29 (41.4)	0.075
27 mm	31 (8.7)	4 (5.6)	0.404	8 (11.4)	4 (5.7)	0.227
29 mm	2 (0.6)	0 (0.0)	0.530	0 (0.0)	0 (0.0)	ND

ND, not determined.

**Table 3:** Postoperative outcomes.

Indicator	Overall Cohort			Propensity Score Matched Cohort		
	Full Sternotomy *n =* 356	Mini-Sternotomy *n =* 70	*p*	Full Sternotomy *n =* 70	Mini-Sternotomy *n =* 70	*p*
Ventilation time, mean ± SD, h	11.7 ± 17.0	9.7 ± 1.7	0.213	11.7 ± 1.4	9.7 ± 1.7	<0.001
Blood loss ≥1000 mL/24 h, *n* (%)	27 (7.6)	1 (1.4)	0.069	2 (2.9)	1 (1.4)	0.559
24-h chest tube drainage, mean± SD, mL	411.9 ± 294.6	256.2 ± 28.6	<0.001	407.6 ± 40.37	256.2 ± 28.6	<0.001
Coagulopathy correction, *n* (%)	87 (24.4)	0 (0.0)	<0.001	14 (20.0)	0 (0.0)	<0.001
Blood product transfusion, *n* (%) Packed red blood cells Platelet concentrates Fresh frozen plasma	78 (21.9) 7 (2.0) 29 (8.1)	12 (17.1) 4 (5.7) 1 (1.4)	0.265 0.096 0.041	15 (21.4) 1 (1.4) 5 (7.1)	12 (17.1) 4 (5.7) 1 (1.4)	0.520 0.172 0.095
ICU stay, mean ± SD, h	83.8 ± 124.8	68.97 ± 6.3	0.305	88.41 ± 20.62	68.97 ± 6.3	0.319
Hospital stay, mean ± SD, days	21.9 ± 18.2	18.3 ± 1.9	0.109	21.9 ± 1.9	18.3 ± 1.9	0.012
Hospital stay after surgery, mean ± SD, days	17.6 ± 16.9	13.06 ± 1.0	0.025	15.2 ± 1.5	13.06 ± 1.0	0.113
30-day mortality, *n* (%)	13 (3.7)	1 (1.4)	0.022	0(0.0)	1 (1.4)	0.316
Resternotomy, *n* (%)	21 (6)	0 (0.0)	<0.001	2(2.9)	1 (1.4)	0.559
Endocarditis, *n* (%)	18 (5.1)	1 (1.4)	0.384	5(7.1)	1 (1.4)	0.095
Wound infection, *n* (%)	19 (5.3)	2 (2.9)	0.645	2(2.9)	2 (2.9)	1,000
Cardiac tamponade, *n* (%)	15 (4.2)	1 (1.4)	0.489	1(1.4)	1 (1.4)	1.000
Acute renal failure, *n* (%)	28 (7.9)	2 (2.9)	0.067	1(1.4)	2 (2.9)	0.559
Other actions, *n* (%) ECMO IABP	2 (0.6) 16 (4.5)	1 (1.4) 0 (0.0)	0.108 0.054	0 (0.0) 0 (0.0)	1 (1.4) 0 (0.0)	0.428
Cardiac rhythm at discharge New atrial fibrillation, *n* (%) New pacemaker, *n* (%)	18 (5.1) 12 (3.4)	3 (4.3) 3 (4.3)	0.071 0.902	3 (4.3) 4 (5.71)	3 (4.3) 3 (4.3)	1.000 0.698
Number of patients treated with: Catecholamine, *n* (%) Morphine, *n* (%)	21 (30.0) 350 (98.31)	4 (5.71) 63 (90.0)	<0.001 0.042	21(30.0) 69 (98.57)	4 (5.71) 63 (90.0)	<0.001 0.029
Morphine dose, mean ± SD, mg	18.56 ± 17.08	8.33 ± 3.58	<0.001	18.56 ± 17.08	8.33 ± 3.58	<0.001
LVEF% at discharge, *n* (%) <30% 30%–49% ≥50%	7 (2.1) 49 (14.7) 278 (83.2)	0 (0.0) 12 (17.4) 57 (82.6)	0.732 0.427 0.428	0 (0.0) 13 (18.6) 57 (81.4)	0 (0.0) 12 (17.4) 57 (82.6)	ND 0.856 0.856

ICU, intensive care unit; ECMO, extracorporeal membrane oxygenation; IABP, intra-aortic balloon pump; LVEF, left ventricular ejection fraction; ND, not determined.

**Table 4:** Factors predicting mortality in patients undergoing aortic valve replacement.

Variables	Relative Risk (RR)	95% CI	*p* Value
Preop hypertension (no vs. yes)	1.717	0.320–9.209	0.528
Preop LVEF (30–49% vs. >50%)	0.156	0.031–0.774	0.023
Aortotomy (transverse vs. J-shaped)	0.761	0.124–4.668	0.768
Type of cardioplegia			
(coronary ostium vs. both)	1.326	0.156–11.301	0.796
(retrograde vs. both)	0.140	0.006–3.494	0.231
Aortic valve type (biological vs. mechanical)	1.059	0.154–7.303	0.953
Coagulopathy correction (no vs. yes)	7.412	1.046–52.508	0.045
